# Development of a Novel IHC Assay for PD-L1 Detection in Non-Small Cell Lung Cancer

**DOI:** 10.3390/biomedicines13102359

**Published:** 2025-09-26

**Authors:** Faye Willett, Marie MacLennan, Sihem Khelifa, Bharathi Vennapusa, Hannah Gautrey, Michael Parkin, Kate R Wilson, Kieran O’Toole, Shubham Dayal, Joseph Chiweshe, Robert Monroe, Fangru Lian

**Affiliations:** 1 Leica Biosystems Newcastle Ltd., Newcastle Upon Tyne NE12 8ET, UK; faye.willett@leicabiosystems.com (F.W.); marie.maclennan@leicabiosystems.com (M.M.); sihem.khelifa@leicabiosystems.com (S.K.); bharathi.vennapusa@leicabiosystems.com (B.V.); hannah.gautrey@leicabiosystems.com (H.G.); michael.parkin@leicabiosystems.com (M.P.); kate.wilson@leicabiosystems.com (K.R.W.); kieran.otoole@leicabiosystems.com (K.O.); 2 Leica Biosystems Richmond Inc., Deer Park, IL 60010, USA; shubham.dayal@leicabiosystems.com (S.D.); joseph.chiweshe@leicabiosystems.com (J.C.); rmonroe01@dhdiagnostics.com (R.M.)

**Keywords:** CAL10, NSCLC, BOND-III, GT 450, PD-L1, immunohistochemistry, tumor proportion score (TPS), SP263

## Abstract

**Background/Objectives:** Programmed cell death-ligand 1 (PD-L1) is one of the key biomarkers for immune checkpoint inhibitors. We are developing a novel PD-L1 CAL10 immunohistochemistry (IHC) assay (Leica Biosystems) on BOND-III staining system and have analyzed its initial performance by comparing it to the PD-L1 SP263 assay (Ventana) assay in a feasibility study. The study objective was to determine the concordance of the Leica Biosystems PD-L1 CAL10 assay with the comparator SP263 assay at the tumor proportion score (TPS) cutoff of ≥50% in non-small cell lung cancer (NSCLC) tissue samples. Additionally, the concordance between the two assays at the TPS cutoff of ≥1% was also evaluated. For informational purposes, we also evaluated the concordance between manual slide reads vs. digital reads (whole slide images generated using the Aperio GT 450) for the CAL10 assay. **Methods:** Two pathologists read and scored the glass slides. The CAL10 PD-L1 assay concordance with the PD-L1 SP263 assay was evaluated by assessing the agreement rates between the two assays. **Results:** The lower bound of the 95% confidence interval (CI) of the overall percent agreement (OPA) at ≥50% cutoff was 86.2%, while for ≥1% TPS cutoff, it was 94.0%, which met the predefined target of a minimum OPA lower bound of the 95% CI value of 85%. **Conclusions:** The Leica Biosystems CAL10 PD-L1 assay has demonstrated comparable performance to the SP263 assay. Additionally, the PD-L1 CAL10 stained glass slides and the corresponding whole side images generated by GT 450 showed comparable concordance rate.

## 1. Introduction

Lung cancer is the leading cause of cancer deaths [1]. In the U.S. alone, in 2025, more than 200,000 new cases of lung cancer will be diagnosed, and 100,000 deaths will be caused due to this disease [2]. Immune checkpoint proteins keep the T-cells in check by inhibiting these cells from attacking their own normal/healthy cells and are considered to be an important target in the treatment of lung cancer. Under normal conditions, these immune inhibitor molecules such as PD-1/PD-L1 function as negative regulators and maintain the balance of the immune system. PD-1 is expressed on the surface of T-cells or other immune cells, and its ligand, PD-L1, is expressed on the surface of antigen-presenting cells (APCs) and other immune cells. Tumor cells can also express PD-L1 and PD-1. PD-1 or PD-L1 on the surface of tumor cells can bind their ligands on T-cells, inducing their death, leading to downregulation of the T-cell response, and helping tumor cells to escape from the host immune response. Tumor cells upregulate these immune inhibitor molecules to evade the immune system, resulting in tumor initiation, progression, and metastasis [3]. The immune checkpoint inhibitor (ICI) blockade of PD1/PD-L1 preserves such T-cells and activates the body’s antitumor immunity.

In 2018, Honjo was awarded the Nobel Prize in Physiology or Medicine for discovering the PD-1 receptor, highlighting the significance of PD-1/PD-L1 signaling pathway immunotherapy [4]. In the past decade, PD-1/PD-L1 ICIs or PD-1/PD-L1-targeted therapy has been shown to improve the overall survival rate in several cancer types, including NSCLC patients [5,6]. PD-1/PD-L1 treatment has been tested in many clinical trials as first, second, or third line of treatment, both as a monotherapy and in combination with other drugs [7,8,9]. Immunotherapies against this receptor–ligand complex block the interaction of the PD-1 receptor and its ligand, PD-L1, preserving the T-cells, thereby allowing these immune cells to mount a response against the tumor.

For both anti-PD-1 as well as anti-PD-L1 treatments, PD-L1 immunohistochemistry (IHC) is used as a standard method for selecting patients for treatment. Assessment of PD-L1 protein expression using IHC is conducted using various scoring criteria. One of them, called combined positive score (CPS) measures PD-L1 expression in immune cells (IC) within the tumor area (macrophages and lymphocytes) as well as in tumor cells (TC). A second scoring method called the tumor proportion score (TPS) assesses the proportion of PD-L1 positively stained tumor cells over the total number of viable tumor cells. As of 2025, the FDA has approved 12 PD-L1 companion diagnostics for immunotherapies, each utilizing different scoring methods [10].

Several studies and trials have used different PD-L1 positivity cutoffs in tumor cells as a criterion for treatment eligibility. Many clinical studies and FDA-cleared or CE IVD-marked companion diagnostics set PD-L1 positivity cutoff ranging from ≥1% to ≥50% [5,11,12,13,14]. We tested the CAL10 clone at both ≥1% and ≥50% TPS cutoffs to evaluate the commonly used thresholds within this feasibility study.

In this article, we are presenting initial results of the PD-L1 (CAL10 clone) assay, which is under development, for use in NSCLC specimens, as well as its performance when assessed by pathologists in the digital pathology realm. In addition to providing an alternate PD-L1 testing option to the healthcare community, this assay could also be a potential candidate for multiplexing and combination therapy.

### Objective

The primary aim of this development study was to measure the concordance of the tested PD-L1 (CAL10) with the comparator SP263 assay at ≥50% TPS cutoff. Also, for exploratory analysis, we performed a ≥1% TPS cutoff staining comparison between the two assays. We targeted a minimum confidence interval (CI) of 85% lower bound of the OPA estimate.

In addition to the PD-L1 CAL10 staining performance analysis, for informational purposes only, the concordance (at ≥50% TPS cutoff and ≥1% TPS cutoff) of CAL10-stained slides between manual and whole slide scanning images was also evaluated. There was no preset target concordance percentage for this analysis.

## 2. Materials and Methods

### 2.1. Inclusion Criteria

The study inclusion criteria for concordance evaluation at ≥50% TPS were as follows: for NSCLC tumor samples, both resection and biopsy cases should be included. Of these specimens, 60–70% should be adenocarcinomas, while 30–40% should be squamous cell carcinomas. Study cases should originate from both primary and metastatic sites, include at least one large cell carcinoma sample, and ensure that at least 10% of the cases are borderline (since the primary objective was to evaluate the concordance at 50% TPS, we did not set any predefined target number for borderline cases at ≥1% TPS cutoff, although the borderline TPS% for ≥1% TPS cutoff was <10% TPS).

There were no set inclusion criteria for the glass slide read vs. digital whole slide image read study, as this analysis was for informational purposes only.

### 2.2. Case Characteristics

Based on the inclusion criteria, we included 136 formalin-fixed paraffin-embedded (FFPE) tissue samples from individual cases of NSCLC. Out of the tested 136 cases, 76 were resection cases and 23 were biopsied cases, while the method of tissue acquisition for the remaining samples was unknown. A total of 88 cases were adenocarcinomas and 43 were squamous cell carcinoma samples, while the status type of the remaining cases was unknown.

Out of the tested 136 cases, 100 were from the primary site, 28 were from the metastatic site, while the status of the remaining cases was unknown. One case of large cell carcinoma was included in the study. Twenty-one cases were borderline (40–60% TPS ranges). A case-wise clinicopathological description is detailed in Table S1.

### 2.3. Screening

All the cases were pre-screened/pre-characterized using BOND RTU PD-L1 (73-10) clone by an enrolling pathologist. Cases with a TPS range of 0–100% were included.

The primary reason for pre-screening the cases with Leica Biosystems 73-10 clone was that the staining protocol used for the screening of this clone produced a similar staining pattern and intensity to the protocol of the test clone, BOND RTU Primary Antibody PD-L1 (CAL10).

### 2.4. Randomization

The stained NSCLC samples included in the study were randomized, and each case was given a unique ID. Two pathologists independently read the paired slide sets and recorded the PD-L1 status (TPS) on a score sheet provided by the sponsor. Once the slide readings were completed, the randomized anonymized IDs were matched with the actual case IDs by the statistician.

### 2.5. Staining

**Figure 1:**
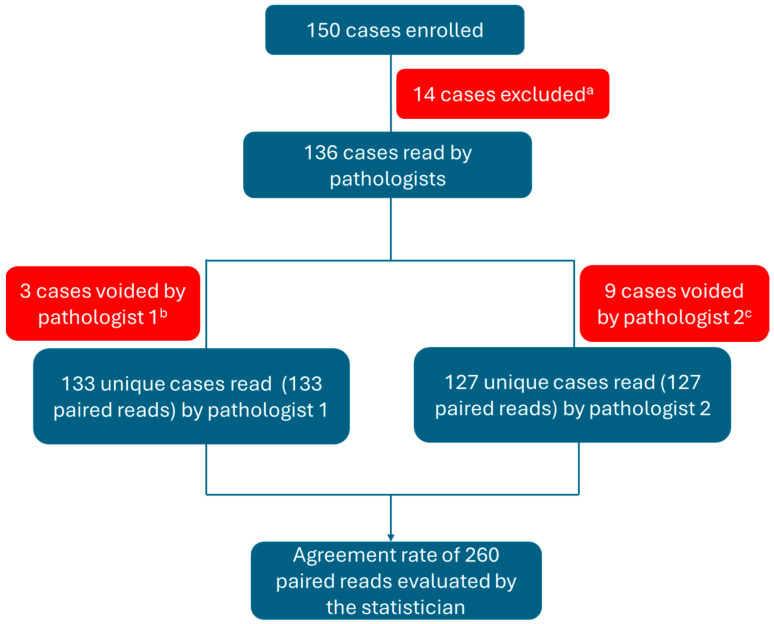
Case distribution schematic. a. Cases were excluded, as one of the staining runs was not completed due to a power failure at the study site. b. Voided due to tissue being washed off or folded. c. Voided due to folded tissue, high background, cytoplasmic blush, shredded tissue, extensive necrosis, folded tissue, or staining in the negative control.

The samples were stained with CAL10 on the BOND-III staining system (using Leica Biosystems ancillaries and consumables), while SP263 samples were processed using the Benchmark Ultra staining system (using Ventana ancillaries, consumables, and recommended protocol). Each case had triplicate glass slides of tissue stained with CAL10, H&E, and an appropriate negative control isotype. Similarly, for the same case, a paired slide set was created that was stained with SP263, H&E, and a manufacturer-recommended negative control isotype. A multi-tissue block containing tonsil and placenta tissues was used as a positive control. The slides were read by two independent pathologists. Figure 1 details the processing of tissue samples.

### 2.6. Scanning

The CAL10-stained glass slides were scanned using the Aperio GT 450 scanner (Leica Biosystems, Vista, CA, USA). After a washout period of 4 months, the whole slide images of the CAL10 stained cases were read by the pathologists. All 136 CAL10 stained glass slides were read by the pathologists; six were voided, and the remaining 130 cases were used to assess the comparability between the two modalities. Since the evaluation of this measurement was not a primary objective, an in-depth statistical calculation of confidence intervals was not performed at either of the cutoffs.

### 2.7. Statistics

For both ≥50% and ≥1% TPS cutoffs, a one-sided, exact, non-inferiority test for a single proportion with a 0.05 type 1 error rate was applied to compare the non-inferiority of the tested CAL10 assay to the SP263 assay.

The concordance or agreement between CAL10 and SP263-stained slides was evaluated against the PD-L1 scoring status (positive or negative) of each stained sample at each cutoff. For ≥50% TPS cutoff, PD-L1 status for a specimen was positive if the TPS was ≥50% and negative if the TPS was <50%. Similarly, for TPS ≥ 1% cutoff, PD-L1 status for a specimen was positive if the TPS was ≥1% TPS and negative if the TPS was <1%.

The paired read agreement rate was calculated using positive percent agreement (PPA), negative percent agreement (NPA), and OPA with 2-sided 95% confidence interval (CI) by the study Biostatistician, applying the Wilson score method. For 50% and 1% TPS cutoffs, the target OPA lower bound 95% CI was set at 85%.

## 3. Results

### 3.1. PD-L1 (CAL10) Assay Showed Comparable Agreement Rates with the SP263 Assay at ≥50% and ≥1% TPS Cutoffs

AT ≥50% TPS cutoff, out of a total 260 paired pooled reads, the OPA was 90.4% with a lower bound CI of 86.2%, while at ≥1% cutoff from the same number of paired slide reads, the OPA was 96.9% with a 94% lower bound. These values met the target OPA lower bound of at least 85%. A detailed analysis is provided in Table 1.

### 3.2. CAL10 Stained Manual and Digital Slide Reads Were Comparable

The pooled analysis for a total of 260 reads (130 unique CAL10 stained cases) for both the study pathologists (see Supplemental Table S2 for each pathologist’s concordance rate) at ≥50% TPS and ≥1% TPS had an OPA of 91.5% (238 agreements/260 reads) and 97.3% (253 agreements/260 reads), respectively. Detailed results of the CAL10 concordance between the two modalities are provided in Table 2.

## 4. Discussion

In this study, we demonstrated that the novel PD-L1 CAL10 assay performance was comparable to the PD-L1 SP263 assay in a development environment.

**Figure 2:**
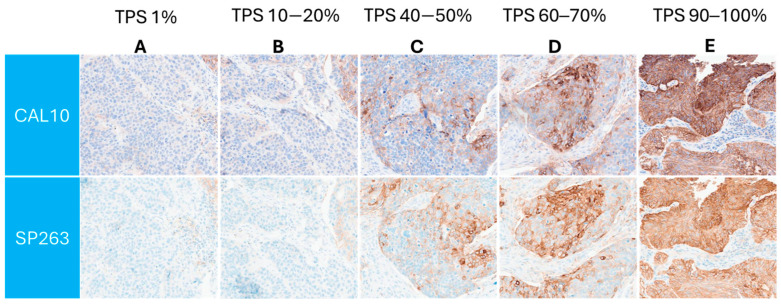
(**A**–**E**): Corresponding TPS expression levels from 1% to 100%. Both assays had similar ranges of expressions in their corresponding chromogenic spectrum. Images produced by the Aperio GT 450 scanner (Magnification: 20×).

Similar PD-L1 membranous staining pattern between both the assays was observed across a range of TPS, as is demonstrated in Figure 2.

Additionally, when the manual light microscopy reads were compared to the GT 450 generated whole slide images, the concordance rate between the two modalities (also in the development environment) was agreeable.

To our knowledge, this is the first feasibility study that assessed the immunohistochemical staining capability of the CAL10 clone on the BOND-III system, while also evaluating its adaptability in DP by digitally scoring the whole slide images and comparing the PD-L1 scoring status to the corresponding glass slides.

The results between the two assays (CAL10 vs. SP263) showed a pooled overall concordance rate of more than 90% across both cutoffs and reaching nearly 97% OPA—which is similar to the studies where the same SP263 assay was compared with other PD-L1 clones, such as the commercially available 22C3 antibody [14], in the same tissue type. Additionally, this interassay variability rate is similar to the SP263 vs. 22C3 agreement rate reported in the Blueprint phase 1 [15] study, where the PD-L1 status was scored using the TPS algorithm—the same scoring algorithm used in this study. Furthermore, the SP263 assay concordance with other assays in that study was around 90% which was consistent with what we observed with the PD-L1 CAL10 assay. Hence, suggesting that the tested CAL10 clone’s preliminary performance is promising.

Previously, Kinstler et al. [16] used the CAL10 clone (Biocare Medical, Zytomed Systems GmbH, Berlin, Germany) and data demonstrated that the CAL10 clone’s PD-L1 expression is comparable to other PD-L1 assays [16]. However, that study used only 10 cases of NSCLC, while the current study, which utilized a CAL10 assay (protocol conceptualized and developed by Leica Biosystems) used 136 NSCLC specimens for the study, providing a larger data set, from which inference regarding the PD-L1 staining could be made more confidently in relation to the comparator assay. Similarly, Mei et al., while scanning the CAL10 slides on the Aperio scanner, showed that in their CAL10 clone (Abcam, Cambridge, UK) PD-L1 positivity was similar to other comparator clones when used in lung cancer. However, in that study, instead of the commonly used whole tissue section of FFPE, tissue microarray (TMA) cores were used [17]. These studies show that the CAL10 clone has been in use for some time, but under different research conditions.

Leica Biosystems has internally developed a PD-L1 CAL10 assay to examine its performance in NSCLC tissue, utilizing Leica Biosystems immunohistochemical staining tools and using TPS, a standardized scoring algorithm. It has been shown in the Blueprint study that interchanging assay-specific variables may impact the staining performance of the antibody [15]. We therefore used the manufacturer-recommended (following Leica Biosystems protocol in development for CAL10 and the comparator’s IFU) staining platforms as opposed to the single staining platform for all the clones used in that study [14], thus allaying any concerns related to the variability in concordance due to the autostainer.

Out of the initially enrolled 150 NSCLC cases, 136 cases were used, as 14 unique cases could not be run on Benchmark Ultra due to a power failure at the study site. Since these specimens were randomly selected for each assay run, any selection bias was negated and had no effect on the overall tissue cohort composition. Moreover, after further investigation and restaining of these cases, it was found that 12 of these NSCLC cases were <50% TPS, and two were voided due to the quality of the tissue. After creating the mock-up table of the best-case scenario (where all cases had 100% agreement rate) and worst-case scenario (where all the agreement rates had 0% agreement), it was found by the study statistician that the inclusion of these additional data points would have minimal effect on the measured endpoints, i.e., NPA, PPA, and OPA. Hence, these cases were excluded from the final analysis.

Out of these 136 stained samples, due to issues such as tissue folding, excessive background, tissue shredding, and cytoplasmic blush in either SP263 or CAL10-stained slides (see Table S4), the first pathologist could read 133 unique cases/slides. Similarly, the second pathologist read 127 slides, leading to a pooled analysis of 260 reads for both assays (see Figure 1). Out of a total of 260 paired reads, 22 paired reads at ≥50% TPS and seven paired reads at ≥1% TPS cutoffs were discordant; the majority of the discordant reads were around the TPS 40–60% (for ≥50% TPS cutoff) or <10% TPS (for ≥1% TPS cutoff) borderline cases. This was not surprising, as borderline staining may be subjective from one pathologist to the other. In this study, we did not give the pathologists an option for a second opinion, which is normally a standard in clinical practice for challenging cases. Although 10% borderline cases in the cohort have been used in other studies [18], increasing the number of challenging cases and subsequently evaluating the concordance rate in totality (of the cohort) or separately (of the borderline cases only) would provide more information on the assay performance.

The study cohort was selected according to the predefined inclusion criteria, which were based on the clinical composition of the NSCLC samples, replicating a real-world setting. For example, we aimed for 30–40% of cases to be squamous cell carcinoma (see inclusion criteria section), and since we were within that range (~32%; 43 squamous carcinoma samples out of 136 total NSCLC samples), it was not deemed necessary to investigate the demographics of the remaining samples.

In addition to the SP263 clone, in a separate mini-method comparison study, we also compared the PD-L1 CAL10 assay to other comparative assays, including 22C3 (pharmDx-Agilent Dako, Agilent Technologies, Inc., Santa Clara, USA) and 28-8 (pharmDx-Agilent Dako, Agilent Technologies, Inc., Santa Clara, USA), on each assay’s manufacturer-recommended staining platforms using NSCLC samples. Of note is the selection of three comparator PD-L1 assays, which was made due to the similarity of the staining pattern amongst the three clones in the pivotal Blueprint study [15]. Again, the CAL10 staining performance was comparable to the other clones (Supplementary Table S5). These early results looked promising, although further analysis with a larger data set is required to validate these findings.

DP is a whole slide imaging diagnostic technique that is garnering attention in the scientific community, specifically due to the pathologist’s accessibility of the digitized slides from any location, including laboratory, home office, etc.; therefore, we wanted to examine how CAL10 clone’s manual readability using glass slides compares to the whole slide image digital reads. Here, we found that the OPA was more than 90% for both cutoffs, reaching up to more than 97% OPA concordance between the conventional light microscopy glass slide scoring and whole slide image digital scoring, which is consistent with the concordance rate between the modalities as observed in other studies [19,20,21]. Majority of the discordance for both the cutoffs was in the borderline cases (see Table S3), which is along the expected pattern of tissue analysis [22].

There were some limiting factors in the study, including the sample size, which could have been larger to confirm the current findings; it was, nonetheless, appropriate for a feasibility assessment. Another limitation was that one of the reading pathologists was also the enrolling pathologist when evaluating the clone’s digital vs. glass slide reads. Although the time difference between enrolling the case and reading the digital image was more than 4 months, which would have minimized any recall bias, and it has been shown by Campbell et al. [22] that a pathologist’s memory recall decreases substantially over time, in the future, we would like to include an enrolling pathologist separate from the reading pathologist for the digital vs. glass slide observation analysis as well. Furthermore, having a higher number of pathologists reading each slide will result in increased data points for comparison—particularly for challenging borderline cases—providing further credence to the observed results. We would also like to evaluate the agreement rate at NSCLC subtype level, such as concordance rate by adenocarcinoma and squamous cell carcinoma types; however, since this was an early feasibility analysis of the assay, further stratification of the concordance rate was out of scope for this study.

Since the aim of this study was to assess the initial performance of the assay, future verification and validation studies—including assessment of inter-pathologist, intra-pathologist, and inter-site reproducibility—will be appropriately sized with the required statistical methods to confirm the PD-L1 CAL10 assay’s findings from the current research study.

As mentioned previously, this Leica Biosystems CAL10 assay has shown encouraging staining performance capability in relation to the selected comparator SP263 assay and has also generated comparable digital images to the manual light microscopy glass slides. These studies were conducted to observe how the assay performs in a smaller setting. Based on these promising data, we plan to further develop the assay through appropriately designed clinical studies with the eventual aim of using the PD-L1 CAL10 assay results for clinical prognosis, thereby improving patient care.

## 5. Conclusions

These initial results suggest that the Leica Biosystems PD-L1 (CAL10) assay is comparable to the SP263 assay and could successfully be adopted for digital pathology assessments upon further development. As mentioned before, this is a development study, and further investigation is required to ascertain the PD-L1 (CAL10) antibody’s diagnostic/clinical utility.

## Figures and Tables

**Table 1:** CAL10 assay staining is comparable to SP263 assay at both ≥50% and ≥1% TPS cutoffs.

**TPS at ≥50% Cutoff**				
**Agreement**	**No. of Agreements**	**No. of Pairs**	**Percent Agreement**	**Confidence Interval**
PPA	108	113	95.6	90.1, 98.1
NPA	127	147	86.4	79.9, 91.0
OPA	235	260	90.4	86.2, 93.4
**TPS at ≥1% Cutoff**				
**Agreement**	**No. of Agreements**	**No. of Pairs**	**Percent Agreement**	**Confidence Interval**
PPA	207	211	98.1	95.2, 99.3
NPA	45	49	91.8	80.8, 96.8
OPA	252	260	96.9	94.0, 98.4

PPA, positive percent agreement; NPA, negative percent agreement; OPA, overall percent agreement.

**Table 2:** CAL10-stained whole slide images were comparable to the glass slides at both ≥50% and ≥1% TPS cutoffs.

**TPS at ≥50% TPS Cutoff**			
**Agreement**	**No. of Agreements**	**No. of Pairs**	**Percent Agreement**
PPA	113	124	91.1
NPA	125	136	91.9
OPA	238	260	91.5
**TPS at ≥1% Cutoff**			
**Agreement**	**No. of Agreements**	**No. of Pairs**	**Percent Agreement**
PPA	199	201	99.0
NPA	54	59	91.5
OPA	253	260	97.3

PPA, positive percent agreement; NPA, negative percent agreement; OPA, overall percent agreement.

## Data Availability

The data will be made available upon request to the corresponding author.

## Supplementary Materials

The following supporting information can be downloaded at https://www.mdpi.com/article/10.3390/biomedicines13102359/s1, Table S1. Clinicopathologic features, Table S2. CAL10 stained digital and manual slide read concordance by each pathologist, Table S3. Manual vs. Digital slide reads—Discordant cases, Table S4. Voided Cases, Table S5. CAL10 assay performance vs. comparators (mini-method comparison study).
