# Supplementary material for: Development of a Novel IHC Assay for PD-L1 Detection in Non-Small Cell Lung Cancer
Source: Biomedicines. 2025 Sep 26;13(10):2359. doi: 10.3390/biomedicines13102359 (PMC12562258; doi:10.3390/biomedicines13102359)
Supplement: Supplementary file 1 [file biomedicines-13-02359-s001.zip › biomedicines-3813511-supplementary.pdf]

Supplementary Materials

Table S1. Clinicopathologic features.

Tissue ID	Sample Type	Sex	Age	Procedure	Collection Site	Grade	T	N	M	Stage
2015/A065	Adenocarcinoma	F	56							
2015/A067	Adenocarcinoma	F	46							
2015/A069	Adenocarcinoma	M	47							
2015/A130	Adenocarcinoma	F	61							
2015/A521	Adenocarcinoma	F								
2015/B161	NSCLC									
2015/B198	NSCLC									
2015/B204	NSCLC									
2015/B555	Adenocarcinoma	M	70							IIIA
2016/D027	Adenocarcinoma	M	73	Resection	Right Upper Lobe	G2	T4	N0	MX	
2016/D041	Adenocarcinoma	F	58	Resection	Left Upper Lobe	G3	T2	N1	MX	
2016/D045	Adenocarcinoma	F	65	Resection	Left Lower Lobe	G3	T2	N2		
2016/D068	Adenocarcinoma	F	69	Resection	Right Upper Lobe	G2	T2	NX	MX	
2016/D101	Adenocarcinoma	F	67	Resection	Left Upper Lobe	G3	T3	N0	MX	
2021/B344	NSCLC	F	52							
2021/B938	Large cell carcinoma	M	77							
2024/B387	Adenocarcinoma	M	73	Resection	LUNG, RUL	G2	T4	N1	MX	IIIB
2024/B388	Squamous cell carcinoma	M	75	Resection	LUNG, RUL	G2	T2	N2	M1	IV
2024/B389	Adenocarcinoma	M	63	Resection	LUNG, RUL	G3	T2a	N0	MX	IB
2024/B390	Adenocarcinoma	M	67	Resection	Lung, LUL	G3	T2a	N1	MX	IIB
2024/B391	Adenocarcinoma	M	74	Resection	LUNG, RUL	G3	T2a	N0	MX	IB
2024/B392	Adenocarcinoma	M	81	Resection	Lung, RLL	G3	T2a	N0	MX	IB
2024/B394	Squamous cell carcinoma	M	61	Resection	LUNG, RML/RLL	G3	T2a	N1	MX	IIB
2024/B395	Squamous cell carcinoma	F	84	Resection	Lung, RLL	G3	T2a	N0	MX	IB

Tissue ID	Sample Type	Sex	Age	Procedure	Collection Site	Grade	T	N	M	Stage
2024/B396	Squamous cell carcinoma	F	76	Resection	Salivary Gland	G2	TX	NX	M1	IV
2024/B399	Adenocarcinoma	M	35	Resection	Brain	G3	TX	NX	M1c	IVB
2024/B400	Adenocarcinoma	F	68	Resection	Lung, Left	G2	T1b	N0	MX	IA2
2024/B401	Squamous cell carcinoma	M	48	Resection	Lymph Node, Cervical	G2	TX	NX	M1b	IVA
2024/B402	Squamous cell carcinoma	M	65	Resection	Lung, LLL	G2	T4	N1	MX	IIIB
2024/B403	Squamous cell carcinoma	M	70	Resection	Lung, LUL	G2	T4	N0	MX	IIIA
2024/B405	Adenocarcinoma	M	52	Resection	Lung, Right	G2	T2b	N0	M0	IIA
2024/B408	Adenocarcinoma	M	70	Resection	Lung, LUL	G2	T2a	NX	MX	IB
2024/B409	Adenocarcinoma	M	57	Resection	Lung, RUL	G2	T2	N1	MX	IIB
2024/B410	Squamous cell carcinoma	M	67	Resection	Lung, RUL	G3	T1a	N0	MX	IA1
2024/B411	Squamous cell carcinoma	M	64	Resection	Lung, LUL	G2	T2	N1	MX	IIB
2024/B412	Adenocarcinoma	M	45	Resection	Lymph Node, Hilar	G2	TX	N1	MX	IIB
2024/B413	Adenocarcinoma	F	42	Resection	Lung, RUL/RML	G2	T4	N2	MX	IIIA
2024/B414	Adenocarcinoma	M	69	Resection	Small Bowel	G3	TX	NX	M1	IV
2024/B415	Adenocarcinoma	M	60	Resection	Brain	G2	TX	NX	M1c	IVB
2024/B417	Squamous cell carcinoma	M	68	Resection	Lung, Left	G2	T2b	N0	MX	IIA
2024/B418	Squamous cell carcinoma	F	60	Resection	Lung, RLL	G3	T2	N0	MX	IB
2024/B420	Adenocarcinoma	F	79	Resection	Lung, RUL	G2	T1	N1	MX	IIB
2024/B421	Adenocarcinoma	F	53	Resection	Lung, LLL	G2	T1	N0	MX	IA
2024/B423	Adenocarcinoma	F	68	Resection	Lung, RUL	G2	T2a	NX	MX	IB
2024/B426	Adenocarcinoma	M	65	Resection	Breast. Left	G3	T2b	N0	M0	IIA
2024/B429	Adenocarcinoma	M	51	Resection	Lung, Right	G3	T2a	N0	M0	IB
2024/B430	Squamous cell carcinoma	F	91	Resection	Soft Tissue, Left Neck	G2	TX	NX	M1c	IVB

Tissue ID	Sample Type	Sex	Age	Procedure	Collection Site	Grade	T	N	M	Stage
2024/B432	Adenocarcinoma	M	60	Resection	Lung, Right	G2	T3	N2	M0	IIIB
2024/B433	Adenocarcinoma	M	58	Resection	Lung, Right	G2	T3	N1	M0	IIIA
2024/B435	Adenocarcinoma	M	71	Resection	Lung, RUL	G3	T2a	N1	MX	IIB
2024/B438	Squamous cell carcinoma	M	81	Resection	Lung, RUL/RLL	G2	T3	N0	M1a	IVA
2024/B440	Adenocarcinoma	M	71	Resection	Lung, RUL	G2	T2a	N1	MX	IIB
2024/B442	Adenocarcinoma	F	62	Biopsy	Lung, Right	G2	T2b	N1	M0	IIB
2024/B443	Adenocarcinoma	M	68	Biopsy	Lung, LUL	G3	T2a	N0	MX	IB
2024/B449	Adenocarcinoma	M	45	Biopsy	Lymph Node, Mediastinum	G3	TX	N2	MX	III
2024/B450	Adenocarcinoma	M	53	Biopsy	Brain	G2	TX	NX	M1c	IVB
2024/B451	Squamous cell carcinoma	M	80	Excision	Lymph Node, Axillary	G2	T2	N3	MX	IIIB
2024/B452	Squamous cell carcinoma	F	76	Biopsy	Lymph Node, Paratracheal	G2	T2	N1	MX	IIB
2024/B453	Adenocarcinoma	M	67	Biopsy	Lung, LUL	G2	T2	N1	MX	IIB
2024/B454	Adenocarcinoma	F	78	Biopsy	Lung, LUL	G3	T2a	NX	MX	IB
2024/B457	Adenocarcinoma	F	81	Biopsy	Lung, RLL	G2	T2	N1	MX	IIB
2024/B458	Adenocarcinoma	M	74	Biopsy	Lung, LLL	G2	T4	N0	MX	IIIA
2024/B459	Adenocarcinoma	M	67	Excision	Lung, Right	G1	T1	N0	MX	IA
2024/B460	Adenocarcinoma	F	60	Biopsy	Brain Occipital	G2	TX	NX	M1c	IVB
2024/B461	Adenocarcinoma	M	55	Biopsy	Brain, Suboccipital	G2	TX	NX	M1c	IVB
2024/B463	Adenocarcinoma	F	58	Biopsy	Lung, Right	G3	T2a	N0	M0	IIIA
2024/B464	Squamous cell carcinoma	M	61	Incisional Biopsy	Lymph Node, Mediastinum	G2	T1a	N1	MX	IIB
2024/B465	Adenocarcinoma	F	52	Incisional Biopsy	Lymph Node, Mediastinum	G3	T2a	N1	MX	IIB
2024/B466	Adenocarcinoma	F	72	Incisional Biopsy	Lymph Node, Mediastinum	G3	TX	N2	MX	III
2024/B469	Squamous cell carcinoma	M	68	Incisional Biopsy	Lymph Node, AZYGOS/MEDIASTINUM	G3	T1a	N1	MX	IIB
2024/B470	Adenocarcinoma	M	68	Incisional Biopsy	Lymph Node, Mediastinum	G3	t1A	N1	MX	IIB

Tissue ID	Sample Type	Sex	Age	Procedure	Collection Site	Grade	T	N	M	Stage
2024/B471	Adenocarcinoma	M	61	Incisional Biopsy	Lymph Node, Mediastinum	G3	T1a	N1	MX	IIB
2024/B472	Squamous cell carcinoma	M	58	Incisional Biopsy	Lymph Node, Mediastinum	G3	T1a	N1	MX	IIB
2024/B474	Squamous cell carcinoma	F	66	Incisional Biopsy	Lymph Node, Mediastinum	G2	T1a	N2	MX	IIB
2024/B475	Squamous cell carcinoma	M	66	Incisional Biopsy	Lymph Node, Mediastinum	G2	T1a	N3	MX	IIB
2024/B476	Squamous cell carcinoma	F	80	Incisional Biopsy	Lung, Right Pleura	G2	TX	N2	MX	III
2024/B477	Adenocarcinoma	F	71	Incisional Biopsy	Lymph Node, Mediastinum	G3	TX	N1	MX	IIB
2024/B566	Squamous cell carcinoma	F	64				T3	N0	MX	IIB
2024/B567	Adenocarcinoma	M	61				T1b	N0	MX	IA
2024/B568	Adenocarcinoma	F	54				T1a	N0	MX	IA
2024/B570	Squamous cell carcinoma	M	65				T1a	N0	MX	IA
2024/B571	Adenocarcinoma	F	55				T2	N1	MX	IIB
2024/B572	Adenocarcinoma	M	62				T2a	N0	MX	IB
2024/B573	Squamous cell carcinoma	M	74				T2b	N1	MX	IIB
2024/B574	Squamous cell carcinoma	M	59				TX	NX	M1	IV
2024/B578	Adenocarcinoma	F	60				T1c	N0	MX	IA3
2024/B579	Squamous cell carcinoma	M	69				T3	N0	MX	IIB
2024/B582	Adenocarcinoma	M	62				T4	NX	MX	IIIA
2024/B585	Squamous cell carcinoma	F	71				T2	N0	MX	IB
2024/B590	Squamous cell carcinoma	F	62				T1c	N0	MX	IA3

Tissue ID	Sample Type	Sex	Age	Procedure	Collection Site	Grade	T	N	M	Stage
2024/B591	Squamous cell carcinoma	M	71				T2a	N0	MX	IB
2024/B595	Adenocarcinoma	F	69				T2b	N0	MX	IIA
2024/B598	Adenocarcinoma	M	81				T1b	N0	MX	IA2
2024/B602	Squamous cell carcinoma	M	73				T2a	NX	MX	IB
2024/B604	Adenocarcinoma	M	61				T2a	N0	MX	IB
2024/B609	Adenocarcinoma	M	77				T2a	N0	MX	IB
2024/B620	Squamous cell carcinoma	F	83				TX	NX	M1B	IVA
2024/B621	Adenocarcinoma	F	71				T2b	N2	MX	IIIA
2024/B623	Adenocarcinoma	M	75				T3	N2	MX	IIIB
2024/B624	Adenocarcinoma	M	56				T2a	N2	MX	IIIA
2024/B626	Adenocarcinoma	F	59				T3	N0	MX	IIB
2024/B627	Adenocarcinoma	M	88				TX	NX	M1	IVA
2024/B631	Squamous cell carcinoma	F	70				T3	N0	MX	IIB
2024/B632	Squamous cell carcinoma	M	67				T2a	N0	MX	IB
2024/B634	Squamous cell carcinoma	M	72				T2a	N0	MX	IB
2024/B671	Adenocarcinoma	M	61	Resection	Lung, LLL	G2	T2a	N0	M0	IB
2024/B673	Adenocarcinoma	M	70	Resection	Lung, Right	G2	T2a	N0	M0	IB
2024/B684	Adenocarcinoma	M	72	Resection	Lung, LEFT	G3	T3	N1	M0	IIIA
2024/B687	Adenocarcinoma	F	62	Resection	Lung, NOS	G1	T2a	NX	MX	IB
2024/B688	Adenocarcinoma	M	66	Resection	Lung, NOS	G2	T2a	NX	MX	IB
2024/B691	Adenocarcinoma	F	75	Resection	Lung, NOS	G2	T2a	NX	MX	IB
2024/B697	Adenocarcinoma	M	70	Resection	Lung, NOS	G2	T2a	N1	MX	IIB
2024/B706	Adenocarcinoma	F	58	Resection	Lung, NOS	G2	T2a	N2	MX	IIIA
2024/B709	Adenocarcinoma	F	73	Resection	Lung, NOS	G3	T2a	N2	MX	IIIA
2024/B711	Squamous cell carcinoma	M	48	Resection	Lung, NOS	G3	T3	N2	M0	IIIB

Tissue ID	Sample Type	Sex	Age	Procedure	Collection Site	Grade	T	N	M	Stage
2024/B715	Squamous cell carcinoma	M	71	Resection	Lung, LLL	G3	T3	N2	M0	IIIB
2024/B716	Squamous cell carcinoma	M	69	Resection	Lung, LLL	G2	T3	N2	M0	IIIB
2024/B718	Squamous cell carcinoma	M	68	Resection	Lung, Right	G2	T3	N2	M0	IIIB
2024/B720	Squamous cell carcinoma	M	81	Resection	Lung, NOS	G3	T3	N2	MX	IIIB
2024/B721	Squamous cell carcinoma	M	55	Resection	Lung, NOS	G3	T3	N2	M0	IIIB
2024/B725	Adenocarcinoma	M	62	Resection	Lung, Right	G2	T2b	N1	M0	IIIA
2024/B730	Adenocarcinoma	M	57	Resection	Lung, Left	G2	T2b	N0	M0	IIA
2024/B731	Adenocarcinoma	M	47	Resection	Lung, Right	G2	T2a	N0	M0	IB
2024/B733	Adenocarcinoma	F	44	Resection	Lung, NOS	G2	T1b	N0	M0	IA
2024/B736	Adenocarcinoma	M	75	Resection	Lung, Right	G3	T3	N2	M0	IIIB
2024/B740	Adenocarcinoma	M	70	Resection	Lung, Right	G2	T3	N2	M0	IIIB
2024/B741	Squamous cell carcinoma	M	67	Resection	Lung, Right	G2	T2b	NX	MX	IIA
2024/B742	Adenocarcinoma	M	58	Resection	Lung, Right	G3	T2b	NX	Mx	IIA
2024/B743	Adenocarcinoma	F	47	Resection	Lung, Left	G2	T2b	NX	MX	IIA
2024/B755	Adenocarcinoma	M	61	Resection	Lung, NOS	G2	T3	NN2	M0	IIIB
2024/B759	Adenocarcinoma	M	39	Resection	Lung, NOS	G2	T1c	N0	M0	IA3
2024/B760	Adenocarcinoma	M	55	Resection	Lung, NOS	G3	T1c	N1	M0	IIB
2024/B771	Adenocarcinoma	M	56	Resection	Lung, NOS	G2	T1b	N0	M0	IA2
2024/B772	Adenocarcinoma	M	62	Resection	Lung, NOS	G3	T1b	N0	M0	IA2
2024/B775	Squamous cell carcinoma	F	53	Resection	Lung, NOS	G2	T2a	NX	MX	IB
2024/B776	Squamous cell carcinoma	M	87	Resection	Lung, NOS	G2	T3	N2	M0	IIIB

RUL, right upper lobe; RML, right middle Lobe; RLL, right lower lobe; LUL, left upper lobe; LLL, left lower lobe; NOS, not otherwise specified; M, male; F, female; G, Grade of cancer cell differentiation; T, Tumor; N, Node; M, Metastasis; I, Clinical Stage/substage of cancer progression.

Table S2. CAL10 stained digital and manual slide read concordance by each pathologist.

**Pathologist A**		**Manual**		
		**≥50% TPS (Pos)**	**<50% TPS (Neg)**	**Total**
Digital	≥50% TPS (Pos)	57	1	58
	<50% TPS (Neg)	5	67	72
	Total	62	68	130
	Agreement (%)	PPA: 91.94	NPA: 98.53	OPA: 95.38
Pathologist B		Manual		
		≥50% TPS (Pos)	<50% TPS (Neg)	Total
Digital	≥50% TPS (Pos)	56	10	66
	<50% TPS (Neg)	6	58	64
	Total	62	68	130
	Agreement (%)	PPA: 90.32	NPA: 85.29	OPA: 87.69
Pathologist A		Manual		
		≥1% TPS (Pos)	<1% TPS (Neg)	Total
Digital	≥1% TPS (Pos)	100	0	100
	<1% TPS (Neg)	0	30	30
	Total	100	30	130
	Agreement (%)	PPA: 100	NPA: 100	OPA: 100
Pathologist B		Manual		
		≥1% TPS (Pos)	<1% TPS (Neg)	Total
Digital	≥1% TPS (Pos)	99	5	104
	<1% TPS (Neg)	2	24	26
	Total	101	29	130
	Agreement (%)	PPA: 98.02	NPA: 82.76	OPA: 94.62

Pos—Positive PD-L1 case; Neg—Negative PD-L1 case; Manual—glass slide read; Digital—whole slide image read.

Table S3. Manual vs. Digital slide reads—Discordant cases.

Pathologist A ≥50% TPS cutoff				
**Tissue ID**	**Digital Read TPS**	**Physical Slides TPS**	**Pathologist A Digital Read Comments**	**Pathologist A Physical Slides Comments**
2024/B771	30	50	Staining intensity is heterogeneous from barely perceptible to 1.5+, located at the periphery of the tumor	Cytoplasmic blush; cover slipping artifact seen on one edge of the tissue
2024/B573	45	50	Heterogeneous staining	DAB entrapment in control tissue, altered tissue with multiple folding may have affected staining
2024/B442	20	50	Heterogeneous tumor with heterogeneous staining	Heterogeneous phenotype
2024/B440	30	55	Heterogeneous basolateral staining with extensive cytoplasmic blush	Basolateral staining in columnar cells, papillary fronds
2024/B415	40	50	Extensive necrosis, crush artifacts and cytoplasmic blush.	Multiple fragments with crush and necrosis, cytoplasmic blush in viable cells, only viable TCs were scored
2021/B938	50	40	Heterogeneous staining from 0+ to 2.5+ within the same tumor nest	Inconsistent staining of TCs within the same tumor nest might be due to suboptimal fixation of this large lymphoid aggregate hosting the tumor cells

Pathologist B ≥50% TPS cutoff			
**Tissue ID**	**Digital Read TPS**	**Physical Slides TPS**	**Pathologist B Digital Read Comments**
2024/B771	35	50	Cytoplasmic and membrane staining.
2024/B602	77.5	30	
2024/B591	60	40	Necrosis with nonspecific staining.
2024/B590	65	40	Weak membrane staining. Challenging read digitally.
2024/B579	55	25	Edge effect. Mixed immune cell and tumor cell staining, weak membrane staining. Borderline case. Challenging read digitally.
2024/B469	50	20	Borderline case.
2024/B442	20	50	

2024/B440	25	55	Cytoplasmic staining makes digital read very challenging.
2024/B438	50	25	Very weak membrane staining, borderline case.
2024/B394	25	95	Dye Trapping.
2024/B392	65	10	
2024/B391	60	30	
2024/B389	15	100	Pigmentation
2021/B938	95	40	
2015/A067	60	30	Pigmentation. Cytoplasmic blush.
2015/A065	45	60	Cytoplasmic blush.
**Pathologist B ≥1% TPS cutoff**			
**Tissue ID**	**Digital Read TPS**	**Physical Slides TPS**	**Pathologist B Digital Read Comments**
2024/B760	1	0.5	Borderline 1% case.
2024/B759	0.5	1	Borderline 1% case. Cytoplasmic and membrane staining.
2024/B631	2.5	0	Necrosis with no specific staining. Mostly immune cell staining. Challenge to separate the immune cells vs. tumor cells digitally.
2024/B403	3.5	0.5	Pigmentation. Weak membrane staining
2024/B401	10	0	Weak membrane staining and cytoplasmic staining. Mixed tumor cells and immune cells.
2024/B388	10	0	Pigmentation
2024/B387	0.5	30	2/3 of the areas with weak membrane staining

Note. For pathologist A, at ≥1% TPS cutoff, there was no discordant data point.

Table S4. Voided Cases.

	**Voided cases-Pathologist 1**
Tissue ID	Pathologist Reason
2015/A069	SP263 void due to washed off tissue
2024/B399	SP263 void due to folded tissue
2024/B470	SP263 void due to folded tissue
	Voided cases-Pathologist 2
2015/A069	SP263 void due to folded tissue
2015/A521	CAL10 void due to very high background and cytoplasmic blush
2016/D041	CAL10 tissue shredded
2016/D101	Extensive necrosis affecting IHC with lots of background
2024/B399	SP263 void due to folded tissue
2024/B423	Very high background with CAL10 and DAB entrapment, cytoplasmic blush
2024/B470	SP263 void due to folded tissue
2024/B582	SP263 void due to folded tissue
2024/B733	Large tissue folding on LBS negative control with specific tumor cell staining, negative control unacceptable

Table S5. CAL10 assay performance vs. comparators (mini-method comparison study).

**50% TPS cutoff**				
		**22C3 pharmDx (Dako Omnis stainer)**		
		≥50% TPS (Pos)	<50% TPS (Neg)	Total
CAL10	≥50% TPS (Pos)	9	4	13
	<50% TPS (Neg)	1	15	16
	Total	10	19	29
	Agreement (%)	PPA: 90.00	NPA: 78.95	OPA: 82.76
		**SP263 Ventana (BenchMark Ultra stainer)**		
		≥50% TPS (Pos)	<50% TPS (Neg)	Total
CAL10	≥50% TPS (Pos)	12	1	13
	<50% TPS (Neg)	0	16	16
	Total	12	17	29
	Agreement (%)	PPA: 100	NPA: 94.12	OPA: 96.55
		**22C3 pharmDx (Autostainer Link 48)**		
		≥50% TPS (Pos)	<50% TPS (Neg)	Total
CAL10	≥50% TPS (Pos)	10	3	13
	<50% TPS (Neg)	2	14	16
	Total	12	17	29
	Agreement (%)	PPA: 83.33	NPA:82.35	OPA: 82.76
		**28-8 pharmDx (Autostainer Link 48)**		
		≥50% TPS(Pos)	<50% TPS (Neg)	Total
CAL10	≥50% TPS (Pos)	12	1	13
	<50% TPS (Neg)	1	13	14
	Total	13	14	27
	Agreement (%)	PPA: 92.31	NPA: 92.86	OPA: 92.59
**1% TPS cutoff**				
**22C3 pharmDx (Dako Omnis stainer)**				
		≥1% TPS (Pos)	<1% TPS (Neg)	Total
CAL10	≥1% TPS (Pos)	23	0	23
	<1% TPS (Neg)	0	6	6

	Total	23	6	29
	Agreement (%)	PPA: 100	NPA: 100	OPA: 100
		**SP263 Ventana (BenchMark Ultra stainer)**		
		≥1% TPS (Pos)	<1% TPS (Neg)	Total
CAL10	≥1% TPS (Pos)	23	0	23
	<1% TPS (Neg)	1	5	6
	Total	24	5	29
	Agreement (%)	PPA: 95.83	NPA: 100	OPA: 96.55
		**22C3 pharmDx (Autostainer Link 48)**		
		≥1% TPS (Pos)	<1% TPS (Neg)	Total
CAL10	≥1% TPS (Pos)	22	1	23
	<1% TPS (Neg)	0	6	6
	Total	22	7	29
	Agreement (%)	PPA: 100	NPA: 85.71	OPA: 96.55
		**28-8 pharmDx (Autostainer Link 48)**		
		≥1% TPS (Pos)	<1% TPS (Neg)	Total
CAL10	≥1% TPS (Pos)	22	0	22
	<1% TPS (Neg)	2	3	5
	Total	24	3	27
	Agreement (%)	PPA: 91.66	NPA: 100	OPA: 92.59

PPA—positive percent agreement; NPA—negative percent agreement; OPA—overall percent agreement; CAL10—PD-L1 assay (test product) that is being compared with other assays; Pos—Positive PD-L1 case; Neg—Negative PD-L1 case.
